# Real-life use of temocillin in the treatment of bone and joint infections due to extended spectrum β-lactamase-producing Enterobacterales

**DOI:** 10.1093/jacamr/dlae171

**Published:** 2024-11-07

**Authors:** Marin Lahouati, Xavier Brousse, Vasco Dias Meireles, Laurine Rignol, Léa Bientz, Fabien Xuereb, Frédéric-Antoine Dauchy

**Affiliations:** CHU de Bordeaux, Service de Pharmacie Clinique, F-33076 Bordeaux, France; Université de Bordeaux, INSERM, Biologie des maladies cardiovasculaires, U1034, F-33600 Pessac, France; CHU de Bordeaux, Centre de référence infections ostéo-articulaires complexes (Crioac GSO) et service des maladies infectieuses et tropicales, F-33076 Bordeaux, France; CHU de Bordeaux, Service de Pharmacie Clinique, F-33076 Bordeaux, France; CHU de Bordeaux, Service de Pharmacie Clinique, F-33076 Bordeaux, France; CHU de Bordeaux, Laboratoire de bactériologie, F-33076 Bordeaux, France; CHU de Bordeaux, Service de Pharmacie Clinique, F-33076 Bordeaux, France; Université de Bordeaux, INSERM, Biologie des maladies cardiovasculaires, U1034, F-33600 Pessac, France; CHU de Bordeaux, Centre de référence infections ostéo-articulaires complexes (Crioac GSO) et service des maladies infectieuses et tropicales, F-33076 Bordeaux, France

## Abstract

**Objectives:**

The aim of this study is to describe the real-life use of temocillin in bone and joint infections (BJI).

**Patients and methods:**

We performed a monocentric retrospective study, including all patients treated by temocillin for a BJI due to extended spectrum β-lactamase-producing Enterobacterales (ESBL-E) between 1 January 2015 and 31 December 2022. Outcomes were evaluated at least 3 months after the end of antimicrobial treatment. Clinical cure was defined as the absence of recurrence of BJI during follow-up among patients who completed at least 7 days of temocillin. If the patient discontinued temocillin due to ineffectiveness, the outcome was considered to be unfavourable. Seventeen patients were treated with temocillin for ESBL-E associated BJI during the study period.

**Results:**

Infections included osteomyelitis of the foot (7/17; 41.2%), femoral osteomyelitis (4/17; 23.5%), disco-vertebral infections (2/17; 11.8%), total knee prosthesis infections (2/17; 11.8%) and total hip prosthesis infections (2/17; 11.8%). All patients except one (*n* = 16) had surgical management of the infection. The main bacteria identified were the *Enterobacter cloacae* complex (*n* = 9) and *Klebsiella pneumoniae* (*n* = 5). The median daily dose was 6 g for a median duration of 42 days (IQR 14–42 days). The median duration of follow-up was 12 months (IQR 5.25–14.5). Overall, 12 patients completed at least 3 months of follow-up, and clinical cure was observed in eight of them (8/12; 66.7%).

**Conclusion:**

So far, this is the first report of BJI successfully treated with temocillin. This suggests that temocillin may be an alternative to treat BJI involving difficult-to-treat Enterobacterales when oral therapy is not available.

## Introduction

Gram-negative bacilli are implicated in 10% of bone and joint infections (BJI).^1^ In cases of difficult-to-treat infections, including extended spectrum β-lactamase-producing Enterobacterales (ESBL-E), carbapenems are sometimes the only treatment available. Although most ESBL-E remain susceptible to carbapenems, the growing reliance on these last-resort antibiotics has led to the increased spread of carbapenemases.^2^ As a result, identifying carbapenem-sparing alternatives has become a priority. Temocillin is a penicillin derived from ticarcillin. Its stability against most β-lactamase, such as ESBL, has led to the proposal of temocillin as an alternative to carbapenems in urinary tract infections caused by ESBL-E.^3^ Moreover, a recent study shown that temocillin has limited impact on the disturbance of the intestinal microbiota.^4^ Furthermore, due to its 24-hour physicochemical stability,^5^ it may be a candidate for outpatient parenteral antimicrobial therapy.^6^ All these characteristics make temocillin an interesting possibility for treating patients with Enterobacterales-associated BJI. However, there are no data on the clinical outcomes of temocillin in BJI. The aim of this study is to describe the real-life use of temocillin in BJI.

## Patients and methods

### Study design

We performed a monocentric retrospective study, including all patients treated with temocillin for a BJI due to ESBL-E between 1 January 2015 and 31 December 2022. Patients were treated in the Department of Infectious and Tropical Diseases of the University Hospital of Bordeaux, a 3000-bed university-affiliated teaching hospital that serves as a referral centre for complex BJIs in south-western France (Crioac GSO). The diagnosis of BJI was based on the 2018 Musculoskeletal Infection Society MSIS^7^ and the 2019 International Working Group on the Diabetic Foot IWGDF^8^ criteria.

### Microbiological

Cultures for bacterial identification were performed from per-operative bone samples. Bacteria were identified by matrix-assisted laser desorption ionization-time of flight mass spectrometry (Bruker Systems). Antimicrobial susceptibility testing was performed by minimum inhibitory concentration (MIC) determination using the BD Phoen™ NMIC-417 panel or by the disc-diffusion method according to the European Committee on Antimicrobial Susceptibility Testing and Antibiogram Committee of the French Society of Microbiology (EUCAST/CA-SFM) guidelines. Using the BD Phoenix™ NMIC-417 panel, the presence of ESBL was confirmed using the MAST^®^ D68C ESBL and AmpC detection set (MAST Group), a combination disc-diffusion test (disc A contains 10 μg of cefpodoxime, disc B has 10 μg of cefpodoxime and clavulanate is the ESBL inhibitor, disc C has 10 μg of cefpodoxime and cloxacillin as the AmpC inhibitor, and disc D has 10 μg of cefpodoxime in combination with both clavulanate and cloxacillin).^9^ When the disc-diffusion method was used, the presence of ESBL was determined by the characteristic aspect of a champagne cork (an expansion of the inhibition zone towards the amoxicillin–clavulanate and cefotaxime, ceftazidime or cefepime discs).

### Data collection

Demographics (age, sex, weight, comorbidities), clinical (type of BJI, implant-associated infection) and microbiological (bacteria identified, susceptibility) data were collected.

Concerning temocillin treatment and previous antimicrobial treatment, characteristics (duration, method and route of administration as well as associated molecules) were collected.

### Outcomes

Outcomes were evaluated at least 3 months after the end of antimicrobial treatment. Clinical cure was defined as the absence of recurrence of BJI during follow-up among patients who completed at least 7 days of temocillin. Failure was defined as the unplanned addition of antimicrobial treatment, the need for additional orthopaedic surgery or death related to the BJI. Outcomes were considered uninterpretable if the patient completed <7 days of temocillin treatment or did not have a follow-up 3 months after the end of antimicrobial treatment.

### Statistical analysis

Continuous data were summarized using the median, interquartile range (IQR = p25-p75), minimum and maximum values. Categorical data were summarized as frequencies and percentages, calculated on the basis of the number of patients with complete data.

### Ethical approval

Our study was conducted following MR-004 reference methodology, relating to the processing of retrospective and prospective personal data implemented in the framework of studies and evaluations in the field of health in France. MR-004 reference methodology obliges the data controller to appoint a Data Protection Officer. Patients were informed when collecting personal data (prior information, opposition right) to comply with GDPR (EU General Data Protection Regulation). Patients did not object to analysis of their data for research.

## Results

Seventeen patients were treated with temocillin for ESBL-E associated BJI during the study period. The median age was 67 years (IQR 59–72) with a median BMI of 26.6 kg/m² (IQR 25–30.8) and 13/17 (76.4%) were male. Median estimated glomerular filtration rate (eGFR) was 88 mL/min (IQR 71.25–106.1). All but one of the patients (*n* = 16) had undergone orthopaedic surgery, and 9/17 patients (52.9%) had history of multiple orthopaedic surgical interventions. Infections included osteomyelitis of foot (7/17; 41.2%), femoral osteomyelitis (4/17; 23.5%), disco-vertebral infections (2/17; 11.8%), total knee prosthesis infections (2/17; 11.8%) and total hip prosthesis infections (2/17; 11.8%). Six patients (35.3%) had implant-associated infections. All patients except one (*n* = 16) had surgical management of the infection (amputation *n* = 7, surgical debridement *n* = 5 and implant removal *n* = 4). Main bacteria identified were *E. cloacae* complex (*n* = 9) and *K. pneumoniae* (*n* = 5). Twelve patients (70.1%) had polymicrobial infections. The different susceptibilities of ESBL-E to antimicrobials are presented in Table 1. Two patients (2/17; 11.8%) had associated EBLS-E bloodstream infection. The median daily dose was 6 g with a median duration of 42 days (IQR 14–42 days). Continuous infusion was prescribed in 8/17 patients (47%). Temocillin was prescribed in combination therapy active on ESBL-E strains (sulfamethoxazole-trimethoprim) in three patients (17.6%). Among all patients treated with temocillin, two had adverse events (rash *n* = 1 and vascular purpura *n* = 1) during treatment, which led to temocillin discontinuation.

**Table 1.** Antibiotics’ susceptibilities to extended spectrum β-lactamase-producing Enterobacterales

Patient	ESBL-E	Antimicrobial susceptibility testing	AMP	AMC	PIP	TIM	TZP	Temocillin	FOX	CXM	LEX	CRO	CAZ	FEP	ATM	ETP	IPM	MEM	TOB	GEN	AMK	NAL	CIP	FOS	SXT
1	*K. pneumoniae*	BMD	R	R	R	R	R	S (8 mg/L)	**R**	R	R	S	R	**S**	I	S	S	S	R	S	I	R	R	S	R
2	*K. pneumoniae*	DD	R	R	R	R	I	S (26 mm)	S	R	R	R	R	R	R	S	S	S	R	R	S	R	R	S	R
3	*K. oxytoca*	BMD	R	R	R	R	I	S (8 mg/L)	S	R	R	R	R	R	R	S	S	S	R	S	S	R	R	S	R
4	*E. cloacae*	BMD	R	R	R	R	R	S (8 mg/L)	R	R	R	R	R	R	R	S	S	S	R	R	S	R	I	S	R
4	*Escherichia coli*	BMD	R	R	R	R	R	S (8 mg/L)	S	R	R	R	R	R	R	S	S	S	R	R	S	R	R	S	R
5	*K. pneumoniae*	BMD	R	R	R	R	R	S (8 mg/L)	S	R	R	R	R	R	R	S	S	S	R	R	S	R	R	S	R
6	*C. sedlakii*	DD	R	R	R	R	S	S (22 mm)	S	R	R	R	R	I	R	S	S	S	R	R	S	R	I	R	R
7	*E. cloacae*	DD	R	R	R	R	S	S (21 mm)	R	R	R	R	R	R	R	S	S	S	R	R	S	R	R	S	R
7	*Citrobacter freundii*	BMD	R	R	R	R	R	R (>32 mg/L)	R	R	R	R	R	R	R	S	S	S	S	S	S	R	R	R	S
8	*E. cloacae*	BMD	R	R	R	R	S	S (4 mg/L)	R	R	R	R	R	S	S	S	S	S	S	S	S	R	I	S	S
9	*K. pneumoniae*	BMD	R	R	R	R	R	S (8 mg/L)	S	R	R	R	R	R	R	S	S	S	R	R	S	R	R	S	R
10	*E. coli*	DD	R	R	R	R	S	S (27 mm)	S	R	R	R	R	R	R	S	S	S	R	R	S	R	R	S	R
11	*E. cloacae*	DD	R	R	R	R	R	S (21 mm)	R	R	R	R	R	R	R	S	S	S	R	S	S	R	R	S	R
12	*K. pneumoniae*	DD	R	R	R	R	I	S (20 mm)	S	R	R	R	R	R	R	S	S	S	R	R	S	R	R	S	R

Abbreviations: AMP, ampicillin; AMC, amoxicillin/clavulanic acid; PIP, piperacillin; TIM, ticarcillin/clavulanic acid; TZP, piperacillin/tazobactam; FOX, cefoxitin; CXM, cefuroxime; LEX, cefalexin; CRO, ceftriaxone; CAZ, ceftazidime; FEP, cefepime; ATM, aztreonam; ETP, ertapenem; IPM, imipenem; MEM, meropenem; TOB, tobramycin; GEN, gentamicin; AMK, amikacin; NAL, nalidixic acid; CIP, ciprofloxacin; FOS, fosfomycin; SXT, trimethoprim/sulfamethoxazole. Antimicrobial susceptibility was determined by BMD, broth microdilution using BD Phoenix™ (MIC, mg/L) or DD, disc diffusion (diameter, mm); susceptible; I, intermediate; R, resistant (according to CASFM 2019/EUCAST).

The median duration of follow-up was 12 months (IQR 5.25–14.5). Three patients did not reach 3 months of follow-up (two patients died during follow-up without clinical failure, neither death was related to infection, and one patient was lost to follow-up). Two patients were not evaluable because they discontinued temocillin before completing a 7-day course (one AE and one patient switched to ertapenem to simplify antimicrobial treatment). Overall, 12 patients were evaluable, and clinical cure was observed in eight of them (8/12; 66.7%).

Baseline characteristics, infection features and outcomes of clinically evaluable patients are reported in Table 2.

**Table 2.** Baseline characteristics, infection features and outcomes of clinically evaluable patients

Number of	Age (years)	Gender	BMI (kg/m^2^)	Comorbidities	BJI	Surgical management before TEM initiation	ESBL-E	TEM duration (days)	Continuous infusion	Associated treatment active against ESBL-E	TEM early discontinuation	Antimicrobial treatment after TEM (duration, days)	Outcomes (last follow-up: months)
1	52	M	23	Diabetes, PAD Hypertension	Osteomyelitis of the foot	Amputation	*K. pneumoniae*	42	Yes	—	No	No	Failure
2	86	M	26	Diabetes, Hypertension	Osteomyelitis of the foot	None	*K. pneumoniae*	7	No	—	Yes (clinical failure)	No	Failure
3	59	M	37	Diabetes	Osteomyelitis of the foot	Amputation	*K. oxytoca*	22	Yes	—	No	No	Favourable (22)
4	55	M	27	Diabetes, PAD Hypertension, Myocardial infarction	Osteomyelitis of the foot	Amputation	*E. cloacae* and *E. coli*	43	Yes	—	No	No	Failure
5	61	M	26	Diabetes, PAD Hypertension	Osteomyelitis of the foot	Amputation	*K. pneumoniae*	17	No	—	Yes (simplified administration regimen)	ETP (28 days)	Favourable (3)
6	33	M	26	None	Femoral osteomyelitis	Amputation	*C. sedlakii*	11	Yes	—	Yes (TEM shortage)	Cefoxitin (28 days)	Favourable (55)
7	72	M	42	Hypertension, Atrial fibrillation	Femoral osteomyelitis	Amputation	*E. cloacae* and *C. freundii*	38	No	SMX-TMP (*C. freundii*)	Yes (AE: rash)	ETP (28 days)	Favourable (13)
8	67	M	28	Diabetes, PAD Hypertension	Femoral osteomyelitis (nail)	Implant removal	*E. cloacae*	56	Yes	—	No	No	Failure
9	85	F	30	Hypertension	Total knee prosthesis infection	Implant removal and spacer	*K. pneumoniae*	42	Yes	—	No	Doxycyclin (suppressive)	Favourable (3)
10	76	M	34	Hypertension	Total knee prosthesis infection	One-step implant exchange	*E. coli*	84	Yes	—	No	No	Favourable (16)
11	70	M	25	COPD	Total hip prosthesis chronic infection	Implant removal followed by multiple debridements	*E. cloacae*	56	No	—	No	No	Favourable (13)
12	61	M	27	Diabetes	Disco-vertebral infection	Debridement	*K. pneumoniae*	42	No	—	No	No	Favourable (13)

Abbreviations: BMI, body mass index; PAD, peripheral artery disease; COPD, chronic obstructive pulmonary disease; ESBL-E, extended spectrum β-lactamase-producing Enterobacterales; ETP, Ertapenem; F, female; M, male; SMX-TMX, Sulfamethoxazole-Trimetoprim; TEM, temocillin.

## Discussion

This is the first study to describe clinical outcomes of temocillin in BJI. Data concerning outcomes of patients treated by temocillin for ESBL-E related infections are limited. Dinh *et al.*^10^ reported outcomes of 113 patients treated by temocillin for ESBL-E infection: the main site of infection was the urinary tract (85.8%) and cure rate was 86.1% at day 28. Only one patient had a BJI in this study, and experimented with treatment failure.

There are several data on management of BJI involving ESBL-E in the literature. Davido *et al*.^11^ reported a success rate of 62.1% in patients with native osteomyelitis due to ESBL-E, and Papadopoulos *et al*.^12^ a success rate of 66.7% in prosthetic joint infection due to multidrug resistant Gram-negative bacteria. In our study, we observed a similar success rate suggesting that temocillin may be considered as an alternative to treat ESBL-E BJI. Temocillin's stability against ESBL, AmpC enzymes and even KPC-producing Enterobacterales,^13^ along with its favourable safety profile and low impact on microbiota, makes it an appealing alternative to carbapenems, especially in BJI.

We report two AE associated with temocillin, which is not negligible. However, several studies have reported a favourable safety profile for temocillin,^14^ and due to the small number of patients we included, it is not possible to draw definitive conclusions about its safety in BJI. Antimicrobial-related AEs are common in patients with BJI,^15^ and therefore, they must be closely monitored, even after hospital discharge.

So far, there have been no data concerning temocillin penetration in bone tissue, but penicillin penetration ratio is usually from 10% to 30%.^16^ Thus, to attain a higher %fT > MIC, continuous infusion and high doses of temocillin (6 g per day) might be preferred if temocillin is used to treat patients with BJI.^17^

This study has several limitations. First, the retrospective character of the study did not allow us to have a long-term follow-up for all patients: two patients had only a 3-month follow-up, and despite initial treatment success, this should be confirmed over a longer period. Moreover, the number of patients included was small, and patients had different types of infection and surgical strategy, making it difficult to evaluate the intrinsic effectiveness of temocillin. Additionally, we considered a 7-day course of temocillin to be the minimum duration of treatment to evaluate outcomes, although this is relatively short. We chose this duration because 7 days was the minimum time before a potential switch to oral treatment. Notably, among the 12 evaluable patients, the median treatment duration with temocillin was 42 days, and only one patient received a 7-day course of temocillin. Last, molecular characterization of ESBL was not routinely performed, so we were unable to provide insights into patient outcomes based on the ESBL group.

Despite the heterogeneity of patients’ clinical characteristics, it seems possible to consider temocillin for complex BJI involving difficult-to-treat Enterobacterales, when oral treatment is not available, to spare carbapenem.

### Conclusion

To date, this is the first report of BJI treated by temocillin. We report a clinical cure rate of 66.7%, suggesting that temocillin may be an alternative to treat BJI involving difficult-to-treat Enterobacterales when oral therapy is not available.
